# Bacterial pathogens associated with external ocular infections alongside eminent proportion of multidrug resistant isolates at the University of Gondar Hospital, northwest Ethiopia

**DOI:** 10.1186/s12886-017-0548-6

**Published:** 2017-08-22

**Authors:** Ergibnesh Getahun, Baye Gelaw, Abate Assefa, Yared Assefa, Anteneh Amsalu

**Affiliations:** 1 Department of Medical Microbiology, School of Biomedical and Laboratory Sciences, College of Medicine and Health Sciences (CMHS), University of Gondar (UOG), P.O. box 196 Gondar, Ethiopia; 2 Department of Ophthalmology, School of Medicine, CMHS, UOG, P.O. box 196 Gondar, Ethiopia

**Keywords:** External ocular infections, Staphylococcus Aureus, MRSA, Multidrug resistance

## Abstract

**Background:**

External ocular infection is a public health problem in Ethiopia. The aim of this study was to determine the prevalence and antimicrobial susceptibility pattern of external ocular bacterial infections.

**Methods:**

A cross sectional study was conducted at the University of Gondar Hospital among patients seeking health services at the Department of Ophthalmology from January to April, 2016. All patients with suspected external ocular infections were examined under slit lamp microscope. External ocular samples were collected using aseptic techniques. All samples were investigated by culture and bacteria were identified using standard methods. Drug susceptibility test was done using the Kirby-Bauer Disk diffusion method according to the guidelines of Clinical and Laboratory Standard Institute (CLSI).

**Result:**

A total of 312 samples were collected and 58.3% were culture positive. The proportion of Gram positive bacterial pathogens was (88%), and *Staphylococcus aureus* (50.3%) was the predominantly isolated pathogen, followed by Coagulase negative *staphylococci* (CoNS) (33.5%) and *Klebsiella* species (4.7%). Conjunctivitis was the dominant clinical feature, but a high positive result for bacterial pathogens was observed among patients with dacryocystitis cases. The Gram positive bacterial isolates were susceptible to ciprofloxacin, chloramphinicol, amoxicillin-clavulanate and ceftriaxone. However, 65% of these Gram positive bacterial pathogens showed resistance to penicillin, ampicillin and amoxicillin. The prevalence of methicillin-resistant *Staphylococcus aureus* (MRSA) infection was 24% and multidrug resistance (MDR) was observed in 87% of the isolated bacteria.

**Conclusion:**

Conjunctivitis was the dominant ophthalmic disease followed by blepharitis. The dominant bacteria species was *S. aureus* and MRSA infection is increasingly prevalent. The overall MDR bacterial pathogen proportion was very high. The high prevalence of MRSA and MDR bacterial pathogens dictate the need for effective prevention as important as for therapies.

## Background

The human eye which is relatively impermeable to most environmental agents is one of the most complex sensory organs of the human body. However, in certain circumstances, infectious agents gain access into the eye, following different routes and cause infection [1]. Trauma, surgery and systemic diseases are among the contributing factors as routes of entry for infectious agents [2].

The most common external ocular infections include conjunctivitis, blepharitis, dacryocystitis, orbital and periorbital cellulitis. Conjunctivitis (red eye) is inflammation of the conjunctiva, and bacterial conjunctivitis could be characterized by mucopurulent discharge and conjunctival hyperemia [3]. Keratitis is an inflammation of the cornea which may lead to corneal ulcer and corneal blindness [4]. Endophthalmitis is infection of the eye that is caused by the entry of exogenous organisms through trauma, surgery or an infected cornea [5], whereas dacrocystitis results from blockage of the lacrimal duct system [6]. Blepharitis is inflammation of the eyelids which could be characterized by redness, itching and greasy or crusty eyelashes [7].

The World Health Organization (WHO) estimated that globally 285 million people were visually impaired of whom 39 million were blind by the year 2010. The report also showed that 82% of the visual impairment, including blindness was avoidable [8]. Uncorrected refractive errors and cataracts were found to be the main causes for 42% and 33% of the visual impairments, respectively. More than 90% of the world’s visually impaired people live in developing countries [9].

Previous studies in different countries reported the prevalence of bacterial pathogens among patients with ocular infections. For example, in Nepal a 76.7% bacterial growth was reported among adult patients suffering from chronic dacryocystitis [10]. In India, a prospective cohort study noted a prevalence of 85.2% bacteria pathogens among cases of ocular infections [6]. Another study conducted in Nigeria reported a 69.2% bacterial isolates from patients with conjunctivitis [11]. The prevalence of bacterial pathogens among patients suffering from external ocular infections was reported as 74.7% at Jimma Hospital, southwest Ethiopia [12].

Different study reports showed higher proportion of Gram positive bacteria among patients suffering from external ocular infections. For example, in Nigeria the prevalence of *S. aureus* and CoNS was reported to be 27.7% and 22.6%, respectively [13]. A cross-sectional study conducted in Libya reported *S. aureus* and *S. epidermidis* as the most common causative agents of anterior blepharitis [14]. In Jimma, Ethiopia, the prevalence of Gram positive cocci among patients with ocular infection was reported as 52% [12]. In a similar study conducted in Hawassa, southern Ethiopia, the prevalence of Gram positive bacterial isolates was 61.5% and the commonest bacterial isolates *S. aureus*, CoNS, and S*. pneumoniae* accounted for 21%, 18.2%, and 14%, respectively [15]. In Gondar, the prevalence of CoN*S* was reported as 29% and that of *S.aureus* 19.4% among patients with dacryocystitis [16].

Antimicrobial resistance among ocular pathogens especially that of *Staphylococcus* species is a worldwide concern [17, 18]. Drug resistance to most groups of antimicrobials is increasing with a decline in the effectiveness of many commonly used topical antimicrobials. Different risk factors are reported for the increasing prevalence of antimicrobial resistant bacteria pathogens of external ocular infection at the community level. Among these factors poor infection control practice plays a major role. The emergence of antimicrobial resistant pathogenic bacteria increases the risk of treatment failure with potentially serious consequences. Early and accurate diagnosis of bacteria caused ocular infections in health care service facilities is required for a proper treatment and case management of external ocular infections. Therefore, the aim of this study was to determine the prevalence of bacterial pathogens, possible risk factors and their antimicrobial susceptibility patterns among patients with external ocular infection.

## Methods

### Study design, area, and period

A cross sectional study was conducted among patients suspected of external ocular infections at the University of Gondar Hospital (UOG), Department of Ophthalmology from January to April, 2016. The hospital is located in North Gondar Zone of the Amhara Region, northwest Ethiopia, and 748 km from Addis Ababa. The UOG hospital is a referral hospital for northwest Ethiopia with more than 400 beds serving a population of about 5 million. The hospital is one of the biggest tertiary level referral and teaching hospitals in the region. It has established an eye care and training center recently. The center serves about 14 million people of several zones by providing medical and surgical treatment for most eye problems.

### Populations

All patients with ocular infections visiting the University of Gondar Hospital during the study were taken as the source population. Patients who had signs and symptoms for external ocular infections and seeking ophthalmic health service in the hospital in the study period were included and patients who had antimicrobial treatment for the last 5 days and/or undergone previous ocular surgery in the last 7 days were excluded from the study.

### Study variables

The prevalence of bacterial pathogens and their antimicrobial susceptibility patterns were used as dependant variables and socio-demographic characteristics such as age, sex, residence, occupation, and educational status as independent variables. Moreover, risk factors for bacteria caused external ocular infections and antimicrobial resistance were determined. Patients were also examined for different ophthalmic clinical characteristics, such as conjunctivitis, keratitis, dacryocystis, and blephteritis of external ocular infections.

### Sample size and sampling technique

The sample size was determined using a single population proportion formula as follows: n = z^2^ p (1-p)/ d^2^; where: n = the number of ophthalmic patients to be involved in this study; Z = Standard normal distribution value at 95% CI, which is 1.96; P = the prevalence of bacterial pathogens among ophthalmic patients previously reported in Gondar which was 60.8% [19]; d = margin of error taken as 5%. Accordingly, the sample size determined was 366, but only 312 patients were included in the study because of time limit.

### Data collection

#### Socio-demographic and clinical data

Socio-demographic characteristics were collected from each participant by a trained ophthalmic nurse using a pretested structured questionnaire. The clinical pictures of external ocular infections of all patients were examined using a slit-lamp bio-microscope and diagnosed by an ophthalmologist.

#### Specimen collection and transport

Specimens from the external part of the eye, such as conjunctiva, eye lid, lacrimal sac and cornea were collected by an ophthalmologist. Conjunctival specimens were collected using a sterile saline moistened cotton swab, applied by passing the swab gently over the lower tarsal and fornix conjunctiva 2-times [19]. In cases of dacrocystitis, specimens were taken by puncture and aspiration of the lacrimal sac. An antiseptic was first applied to the area of puncture, and then the lacrimal sac was punctured in the area below the medial canthal ligament [20]. Specimens from corneal ulcers or keratitis were collected with the help of the slit lamp microscope. Briefly, a local topical anesthetic (Lidocine with 10% normal saline) was applied. After 3–5 min of draining of the anesthetic, purulent material was removed by a sterile cotton swab and discarded. The edge of the ulcer was firmly scraped using Bard Parker blade and several scrapings were collected. In the case of blephteritis, discharge from the margin of the eyelid was collected using cotton swabs and placed into a sterile tube. All the swabs were finally immersed in a tube that had 3 ml brain heart infusion (BHI) [21] and transported to the diagnostic laboratory of the University of Gondar Hospital for investigation.

### Laboratory methods

#### Culture and identification of bacterial pathogens

Each specimen was inoculated on a Blood agar plate (BAP), Chocolate agar plate (CAP), MaCconkey agar (MAC) and Manitol salt ager (MSA) culture media (Oxoid Ltd. Basingstoke, Hampshire, UK) using sterile wire loops and incubated at 37 °C for 48 h. The CAPs were incubated within a candle-jar to facilitate CO_2_ tension. After 24 h of incubation, the plates were examined for bacterial pathogen growth, and plates with no growth were re-incubated for further 48 h. For each plate that demonstrated mixed bacterial colony growth, subculture was done following the SOPs of the hospital laboratory. Identification of bacterial pathogens was made initially by Gram stain and colony morphology followed by biochemical tests. Catalase, coagulase and bacitracin tests were applied to identify and differentiate Gram positive cocci, while biochemical tests, such as triple sugar iron agar (TSI), citrate utilization, lysine decarboxylase agar (LDC), urease and indole tests were used to identify Gram negative bacterial pathogens [22, 23].

#### Antimicrobial susceptibility test

Antimicrobial susceptibility test was carried out on each identified bacterium using disc diffusion method on Mueller Hinton agar (MHA) (Oxoid Ltd. Basingstoke, Hampshire, UK). However, the antimicrobial sensitivity test for fastidious bacterial pathogens was conducted on MHA medium containing 5% defibrinated sheep blood. Briefly, 3–5 bacterial colonies of the test organism were picked and emulsified in 5 ml of nutrient broth and mixed gently. To standardize the inoculums density for a susceptibility test, a 0.5 McFarland standard solution was used. The plates were then inoculated by streaking the swab over the entire agar surface and the antimicrobial impregnated disks were placed using sterile forceps on the agar surface, incubated at 37 °C for 24 h, and the zone of inhibition was determined. The zone diameters were interpreted according to the Clinical and Laboratory Standards Institute (CLSI) guideline as susceptible (S), intermediate (I) or resistant (R) [24].

#### Quality control

The reliability of the findings was guaranteed by implementing quality control (QC) measures throughout the whole process of the laboratory work. All materials, equipment, and procedures were adequately controlled. The questionnaire was prepared in English, translated to Amharic and then retranslated to English to check for consistency. Data on socio-demographic characteristics and eye related medical history were collected by a trained ophthalmic nurse. All specimens were collected following standard operating procedure (SOPs) for external ocular specimen collection. The sterility of culture media was ensured by incubating un-inoculated media from each batch. The performances of all prepared culture media were checked by inoculating standard-strains, such as *Escherichia co*li (ATCC 25922), *Staphylococcus aureus* (ATCC 25923) and *Pseudomonas aeruginosa* (ATCC 27853) obtained from the Ethiopian Public Health Institute, Addis Ababa, Ethiopia [22, 24]. The qualities of biochemical tests were also checked by these reference strains. Double data entry system was used to maintain data entry quality.

#### Data analysis and interpretation

Data was checked for completeness, coded, and first entered in to EPI-info version 7, and then rechecked and transferred to the Statistical Package for Social Science (SPSS) version 20 for analysis. Bivariate and multivariate logistic regression analyses were used to assess the possible risk factors for bacteria caused external ocular infections. Descriptive statistics were also used to explain antimicrobial susceptibility patterns. Odds ratio (OR) with confidence interval were computed to assess the presence and degree of association between dependent and independent variables. *P*-value <0.05 at 95% CI was considered statistically significant.

## Results

### Socio-demographic characteristics of participants

A total of 312 patients with external ocular infection were included. The majority of the participants were males (168; 53.8%) and the median age was 43 years (IQR = 42.5 years). About one-third of the patients were over 60 years of age and most of them (56.7%) were rural dwellers. Data on occupation showed that 26.9% were farmers, 25.3% house wives, 17.3% students, 10.9% pre-school children, 8.3% civil servants; others accounted for 2%. The majority (61.9%) of the patients had no formal education, 24.4% had primary and secondary schooling; only 13.8% had diploma and above.

### Possible risk factors and clinical findings

The data showed that 215 (68.9%) patients had previous history of ocular infection, but only 14.7% had history of ophthalmic surgery. One hundred seventy-two (55.1%) patients had illness for more than 2 weeks and 33.3% for less than 2 weeks (*n* = 107). The data also showed that 81.7% (*n* = 255) of the patients had no history of ocular trauma. On the other hand, 174 patients had previous history of antimicrobial therapy of whom 33.3% were culture positive for different bacterial pathogens. Multivariate logistic regression analysis showed that an acute infection with duration of 2–4 weeks and previous use of antimicrobials were significantly associated with culture positive bacterial infection of the eye (*P* = 0.028 and *P* = 0.014, respectively) (Table 1).

**Table 1:** Possible risk factors and their association with the prevalence of bacterial infection among patients with external ocular infection at University of Gondar hospital, Northwest Ethiopia, 2016

Variables		Culture results			COR (95% CI)	AOR(95% CI)	*P*-value
		n (%)	-ve n (%)	+ve n (%)			
Previous ocular infection	Yes	215(68.9)	81(37.7)	134(62.3)	1.69(1.041–2.74)		
	No	97(31.1)	49(50.5)	48(49.5)	1		
Previous use of antimicrobials	Yes	147(47.1)	49 (33.3)	98(66.7)	1.93(1.219–3.05)	1.84(1.132–2.97)	0.014
	No	165(52.9)	81(49.1)	84(50.9)	1	1	
History of surgery	Yes	46(14.7)	19(41.3)	27(58.7)	1.02(0.539–1.92)		
	No	266(93.6)	111(41.7)	155(58.3)	1		
Duration of present illness	<1 week	107(34.3)	52(48.6)	55(51.4)	1	1	
	2–4 weeks	33(10.6)	9(27.3)	24(72.7)	2.52(1.072–5.93)	2.72(1.116–6.62)	0.028
	>4 weeks	172(55.1)	69(40.1)	103(59.9)	1.41(0.868–2.29)	1.26(0.754–2.12)	0.34
Ocular trauma	Yes	57(18.3)	30(52.6)	27(47.4)	1		
	No	255(81.7)	100(39.2)	155(60.8)	1.72(0.967–3.068)		

*n* number of patients, *−ve* negative, *+ve* positive, *COR* crude odds ratio, *AOR* adjusted odds ratio

Out of the clinical features of ophthalmic patients, conjunctivitis, blepharitis, dacrocystitis, keratitis, and blepharo-conjunctivitis constituted 56.4%**,** 35.3%, 5.1%, 2.2%, and 1%, respectively. Among the 176 conjunctivitis cases, 48.9% were culture positive for bacterial pathogens and 4 patients had mixed bacterial growth. On the other hand, among the 110 blepharitis cases, 67.3% were culture positive and 4 patients also had mixed bacterial pathogens. Among patients with dacrocystitis, fourteen out of 16 (87.5%) were culture positive for different bacterial pathogens. In this study, 41.7% of the ophthalmic patients were culture negative for bacterial pathogens (Tables 2).

**Table 2:** Distribution of bacterial isolates among different clinical features of external ocular infections at University of Gondar hospital, Northwest Ethiopia, 2016

Bacteria species	Clinical features with total bacteria isolated					
	Conjunctivitis (*n* = 90) n (%)	Blepharitis (*n* = 79) n (%)	Dacryocystitis (*n* = 14) n (%)	Keratitis (*n* = 6) n (%)	Blepharo-conjunctivitis (*n* = 2) n (%)	Total (*n* = 191) n %
*S. aureus*	46(51.1)	40(50.6)	7(50)	3(50)	0	96(50.3)
CoNS	28(31.1)	26(32.9)	7(50)	2(33.3)	1(50)	64(33.5)
*S. pyognes*	3(3.3)	4(5.1)	0	0	1(50)	8(4.2)
*Klebsiella* spp*.*	5(5.6)	4(5.1)	0	0	0	9(4.7)
*E.coli*	4(4.4)	1(1.3)	0	1(16.7)	0	6(3.1)
*Serratia* spp.	0	2(2.5)	0	0	0	2(1.0)
*Citrobacter* spp.	1(1.1)	0	0	0	0	1(0.5)
*Proteus* spp.	2(2.2)	0	0	0	0	2(1.0)
*Entrobacter* spp.	1(1.1)	2(2.5)	0	0	0	3(1.6)
Total *n* = 191	90(47.1)	79(41.4)	14(7.3)	6(3.1)	2(1)	191(100)

### Prevalence of bacterial pathogens

Out of the 312 external ocular specimens taken for bacteriological evaluation, 182 (58.3%) were culture positive. Higher culture positive result (62.5%) was observed among female patients. Patients aged 15–29 and 30–44 years also showed higher culture positive results like 67.2% and 60.8%, respectively. The proportions of culture positive cases were nearly similar among rural and urban dwellers by representing 58.8% and 57.8%, respectively. In this study, the majority of the patients (95.6%; *n* = 174) had single bacterial isolates while only 8 (4.4%) demonstrated mixed bacterial isolates. A total of 191 bacteria pathogens were isolated either singly or in mixed cases. The proportion of Gram positive bacterial pathogens was 88% (*n* = 168/191). *S. aureus* was the dominant bacterial pathogen (50.3%) followed by coagulase negative *Staphylococci* (CONS) (33.5%). On the other hand, the proportion of Gram negative bacterial isolates was 12% (*n* = 23/191) and that of *Klebsiella* species, *E. coli* and *Enterobacter* species 4.7%, 3.1% and 1.6%, respectively. The predominant bacteria isolated from conjunctivitis cases forming the proportions indicated were *S.aureus* (51*.*1%), CoNS (31.1%), and *Klebsiella* species (5.6%). Similarly, *S. aureus* (50.6%) was the most frequently isolated bacteria followed by CoNS (32.9%) among blepharitis cases. In the cases of dacryocystitis, *S.aureus* and CoNS accounted for 50% each. Among patients suffering from keratitis, only *S.aureus* (*n* = 3) and CoNS (*n* = 2) were isolated (Table 2).

### Antimicrobial susceptibility patterns of gram positive bacterial isolates

Out of 168 Gram positive bacterial isolates, 99.4%, 92.3%, 91.2%, 87.5%, 86.9%, 83.3%, and 85.7% were susceptible to cefepime, clindamycin, ciprofloxacin, chloramphinicol, cotrimozaxole, amoxicillin-clavulanate, and ceftriaxone, respectively. However, significant proportions of Gram positive cocci were resistant to penicillin (91.7%), ampicillin (86.9%), amoxicillin (86.9%), and ceftazidime (81%). Among the *S. aureus* isolates, more than 80% were susceptible to cefepime, ciprofloxacin, clindamycin, cotrimozaxole, chloramphinicol, ceftriaxone and amoxicillin-clavulanate. Nevertheless, higher proportions of the *S. aureus* isolates were resistant to penicillin (96.9%), ampicillin (92.7%), amoxicillin (92.7%), and ceftazidime (86.5%). In this study, the prevalence of MRSA, determined based on the resistance pattern of cefoxitin, was 24% (Table 3).

**Table 3:** Antimicrobial susceptibility pattern of Gram positive bacterial isolates of external ocular infections at University of Gondar hospital, Northwest Ethiopia, 2016

Antimicrobials	Gram positive bacteria S or R n (%)							
	*S.aureus,n* = 96		*CoNS,n* = 64		*S. pyogenes,n* = 8		Total, *n* = 168	
	S (%)	R (%)	S(%)	R(%)	S(%)	R(%)	S(%)	R(%)
SXT	89(92.7)	7(7.3)	49(76.6)	15(23.4)	8(100)	0	146 (86.9)	22 (13.1)
TE	70(72.9)	26(27.1)	43(67.2)	21(32.8)	5(62.5)	3(37.5)	118(70.2)	50(29.8)
P	3(3.1)	93(96.9)	3(4.7)	61(95.3)	8(100)	0	14(8.3)	154(91.7)
C	85(88.5)	11(11.5)	54(84.4)	10(15.6)	7(77.5)	1(12.5)	147(87.5)	21(12.5)
E	69(71.9)	27(28.1)	37(57.8)	27(42.2)	8(100)	0	114(67.9)	54 (32.1)
DA	89(92.7)	7(7.3)	61(95.3)	3(4.7)	5(62.5)	3(37.5)	155(92.3)	13(7.7)
CXT	73(76)	23(24)	40(62.5)	24(37.5)	-	-	113(70.6)	47(29.4)
CRO	75(78.1)	20(20.8)	53(82.8)	11(17.2)	8(100)	0	136(81.0)	32(19.0)
CIP	89(92.7)	7(7.3)	57(89.1)	7(10.9)	-	-	146(91.2)	14(8.8)
CFP	95(99)	1(1)	64(100)	0	8(100)	0	167(99.4)	1(0.6)
CN	69(71.9)	27(28.1)	47(73.4)	17(26.6)	-	-	116(72.5)	44(27.5)
AMP	7(7.3)	89(92.7)	7(10.9)	57(89.1)	8(100)	0	22(13.1)	146(86.9)
AML	7(7.3)	89(92.7)	7(10.9)	57(89.1)	8(100)	0	22(13.1)	146(86.9)
AMC	79(82.3)	17(17.7)	53(82.8)	11(17.2)	8(100)	0	140(83.3)	28(16.7)
CAZ	13(13.5)	83(86.5)	11(17.2)	53(89.1)	8(100)	0	32(19.0)	136(81.0)
OX	69(71.9)	27(28.1)	40(62.5)	24(37.5)	-	-	109(68.1)	51(31.9)

*SXT* Cotrimoxazole, *TE* Tetracycline, *P* Penicillin, *C* Chloramphenicol, *E* Erythromycin, *DA* Clindamycin, *CXT* cefoxitin, *CRO* Ceftriaxone, *CIP* Ciprofloxacin, *CFP* cefepime, *CN* Gentamicin, *AMP* Ampicillin, *AML* amoxicillin, *AMC* Amoxicillin-clavulanate, *CAZ* ceftazidime, *OX* oxacillin, *S/R* Sensitive or resistant

### Antimicrobial susceptibility patterns of gram negative bacterial isolates

A total of 23 Gram negative bacteria were identified. The drug susceptibility pattern of these bacteria showed that 91.3% were susceptible to cefepime and 87% for ciprofloxacin, cefoxitin and amoxicillin-clavulanate. On the other hand, more than 78.3% of the Gram negative bacterial isolates were susceptible to cotrimoxazole, ceftazidime, and ceftriaxone together with more than 65% isolates susceptible to tetracycline and chloramphinicol. However, 56.5%, 34.8%, and 30.4% of the Gram negative bacterial isolates were resistant to ampicilin and amoxicillin, tetracycline, and chloramphinicol, respectively. The drug susceptibility pattern of *Klebsiella* species showed 100% resistance to ampicilin and amoxicillin**.** Moreover, more than 30% of *Klebsiella* species were also resistant to cotrimozaxole, tetracycline, ceftriaxone, gentamicin, chloramphinicol and ceftazidime. Nevertheless, more than 75% of the *Klebsiella* species isolates were susceptible to amoxicillin-clavulanate, ciprofloxacin, cefoxitin, and cefepime. The drug susceptibility patterns of *E. coli* showed that all isolates were susceptible to cotrimozaxole, 83.3% to ampcillin, ceftriaxone, cefoxitin, ciprofloxacin, ceftazidime, and 66.7% to tetracycline and chloramphenicol (Table 4).

**Table 4:** Antimicrobial susceptibility pattern of Gram negative bacterial isolates of external ocular infections at University of Gondar hospital, Northwest Ethiopia, 2016

Antimicrobials	Gram negative bacteria S, or R, n (%)													
	Klebsiella spp. (*n* = 9)		*E. coli* (*n* = 6)		Entrobacter spp. (*n* = 3)		Seratia spp. (*n* = 2)		Proteus spp. (*n* = 2)		*Citrobacter* spp. (*n* = 1)		Total (*n* = 23)	
	S (%)	R (%)	S (%)	R (%)	S (%)	R (%)	S (%)	R (%)	S (%)	R (%)	S (%)	R (%)	S (%)	R (%)
SXT	6(66.7)	3(33.3)	6(100)	0	2(66.7)	1(33.3)	1(50)	1(50)	2(100)	0	1(100)	0	18(78.3)	5(21.7)
TE	6(66.7)	3(33.3)	4(66.7)	2(33.3)	1(33.3)	2(66.7)	2(100)	0	1(50)	1(50)	1(100)	0	15(65.2)	8(34.8)
CAZ	5(55.6	4(44.4)	5(83.3)	1(16.7)	3(100)	0	2(100)	0	2(100)	0	1(100)	0	18(78.3)	5(21.7)
C	5(55.6)	4(44.4)	4(66.7)	2(33.3)	3(100)	0	2(100)	0	1(50)	1(50)	1(100)	0	16(69.6)	7(30.4)
CIP	7(77.8)	2(22.2)	5(83.3)	1(16.7)	3(100)	0	2(100)	0	2(100)	0	1(100)	0	20(87.0)	3(13)
CXT	7(77.8)	2(22.2)	5(83.3)	1(16.7)	3(100)	0	2(100)	0	2(100)	0	1(100)	0	20(87.0)	3(13)
CRO	6(66.7)	3(33.3)	5(83.3)	1(16.7)	2(66.7)	1(33.3)	1(50)	1(50)	2(100)	0	1(100)	0	17(73.9)	6(26.1)
CFP	7(77.8)	2(22.2)	6(100)	0	3(100)	0	2(100)	0	2(100)	0	1(100)	0	21(91.3)	2(8.7)
CN	6(66.7)	3(33.3)	4(66.7)	2(33.3)	3(100)	0	2(100)	0	2(100)	0	1(100)	0	18(78.3)	5(21.7)
AMP	0	9(100)	3(50)	3(50)	2 (66.7)	1(33.3)	2(100)	0	2 (100)	0	1(100)		10(43.5)	13(56.5)
AML	0	9(100)	3(50)	3(50)	2(66.7)	1(33.3)	2(100)	0	2(100)	0	1(100)	0	10(43.5)	13(56.5)
AMC	8(88.9)	1(11.1)	5(83.3)	1(16.7)	2(66.7)	1(33.3)	2(100)	0	2(100)	0	1(100)	0	20(87.0)	3(13.0)

*SXT* cotrimoxazole, *TE* Tetracycline, *CAZ* ceftazidime, *C* Chloramphenicol, *CIP* Ciprofloxicin, *CXT* cefoxitin, *CRO* Ceftriaxone, *CFP* cefepime, *CN* Gentamicin, *AMP* Ampcillin, *AML* amoxicilline, *AMC*Amoxicillin-clavulanate

### Patterns of multidrug resistance among bacterial isolates

In this study, antimicrobial resistance was observed in the majority of the bacterial species isolated from external ocular infection. However, 8 bacterial pathogens demonstrated no antimicrobial resistance to any of the antimicrobials tested. About 21% of the *S .aureus* isolates were resistant to 8 antimicrobials, 19.8% for 5 antimicrobials, and 27% for 4 antimicrobials. In this study, there was no *S .aureus* bacterial isolate susceptible to all antimicrobials. On the other hand, 37.5% of the CoNS bacterial isolates showed antimicrobial resistance to 8 antimicrobials but only 1 bacterial isolate was susceptible to all antimicrobials. The drug resistance pattern of *S. pyogens* was by far lower that only 3 out of the 8 isolates showed resistance to tetracycline and clindamycin. Among the Gram negative bacteria, *Klebsiella* species antimicrobial resistance pattern was as low as for 2 antimicrobials and as high as for 8 antimicrobials. Antimicrobial resistance was also observed in 4 out of the 5 *E. coli* isolates, and all the 3 *Entrobacter* species isolates showed resistance for more than 2 antimicrobials. The overall MDR pattern (resistance to three or more antimicrobials) among bacterial isolates taken from external ocular infection was 87% (*n* = 166/191). In this study, the proportion of bacterial isolates susceptible to all antimicrobial agents tested was 4.2% (*n* = 8/191) (Table 5).

**Table 5:** Multidrug resistance patterns of bacterial isolates from external ocular infection at University of Gondar hospital, Northwest Ethiopia, 2016

Types of bacteriaisolates	Total bacteria n (%)	Antimicrobial resistance pattern								
		R0 n (%)	R1 n (%)	R2 n (%)	R3 n (%)	R4 n (%)	R5 n (%)	R6 n (%)	R7 n (%)	R8 n (%)
*S.aureus*	96(50.3)	0	0	3(3.1)	2(2.1)	26(27.1)	19(19.8)	15(15.6)	11(11.5)	20(20.8)
CoNS*	64(33.5)	1(1.6)	1(1.6)	2(3.1)	2(3.1)	8(12.5)	20(31.3)	4(6.3)	2(3.1)	24(37.5)
*S.pyogn*	8(4.2)	2(25)	5(62.5)	1(12.5)	0	0	0	0	0	0
Klebsella spp*.*	9(4.7)	0	0	1(11.1)	1(11.1)	2(22.2)	2(22.2)	2(22.2)	0	1(11.1)
*E.coli*	6(3.1)	2(33.3)		1(16.7)	1(16.7)	0	2(33.3)	0	0	0
Entrobacter spp.	3(1.6)	0	0	2(66.7)	1(33.3)	0	0	0	0	0
Serratia spp.	2(1)	1(50)	1(50)	0	0	0	0	0	0	0
Proteus spp.	2(1)	1(50)	0	0	1(50)	0	0	0	0	0
Citrobacter spp.	1(0.5)	1(100)	0	0	0	0	0	0	0	0
Total	191(61.2)	8(4.2)	7(3.7)	10(5.2)	8(4.2)	36(18.8)	43(22.5)	21(11)	13(6.8)	45(23.6)

CoNS* = Coagulase negative *staphylococci;R0 = sensitive to all antimicrobials;R1 = resistant to 1antimicrobial; R2 = resistant to2antimicrobials;R3 = resistant to 3antimicrobials;R4 = resistant to 4antimicrobials;R5 = resistant to 6antimicrobials;R7 = resistant to7antimicrobials;R8 = resistant to 8antimicrobials; Spp*. *= species*

## Discussion

External ocular infections are the leading causes of morbidity in developing countries. Ocular bacterial infections can cause a series of symptoms and signs, such as the formation of pus, conjunctival hyperemia, lid edema, and even visual impairment [25]. Pathogens that cause ocular infection are generally exogenous. However, in certain circumstances they gain accesses to enter the eye and cause infection. The overall prevalence of bacterial pathogens that caused external ocular infection in the present study was 58.3%. This is comparable to previous reports in the same area, 54.2% [26] and 60.8% [27]. Another recent report from Dessie, northeastern Ethiopia documented a 59.4% prevalence of bacterial pathogens among ophthalmic patients [28]. On the other hand, a relatively higher prevalence (74.7%) of bacteria caused eye infection was reported in Jimma, southwestern parts of Ethiopia [12] and a relatively lower prevalence (48.8%) was reported in Hawassa [15].

In the present study, previous ocular infection, previous use of antimicrobials, history of surgery, duration of the present illness and history of ocular trauma were considered as possible risk factors for bacteria caused external ocular infection. However, the data showed a statistically significant association between history of antimicrobial use and duration of illness of between 2 to 4 weeks with bacteria-caused ophthalmic infection. The major risk factors for bacteria caused ocular infections are surgical and nonsurgical trauma and use of contact lenses [29, 30]. The data of the present study showed that 26.9% of the patients were farmers by occupation. The risk of agricultural predominance and vegetative corneal injury in bacterial keratitis increase susceptibility to corneal infection [31]. In Gujarat, Western India, a study conducted on 200 cases described predominant outdoor agricultural activity as the principal causative factor for corneal injury [32].

The clinical features of the ophthalmic patients in Gondar showed that 56.4% had conjunctivitis, 35.3% blepharitis, 5.1% dacrocystitis, 2.2% keratitis, and 1% blepharo-conjunctivitis. In Hawasa, Amsalu et al. [20] reported a proportion of 49.8%, 19.6%, 11.0%, 6.8%, 3.8%, 2.5%, and 2.1% for conjunctivitis, blepharitis, keratitis, dacrocystitis, external hordeolum, blepharo-conjunctivitis, and lid abscess, respectively. In Dessie, Northeastern Ethiopia, Shiferaw et al. [28] reported a prevalence of 43.1% for conjunctivitis and 29.4% for blepharitis. On the other hand, blepharo-conjunctivitis was found the dominant type clinical feature of external ocular infection at Jimma University hospital, southwest Ethiopia [12].

In the present study, among 176 conjunctivitis cases, 48.9% were culture positive for bacterial pathogens but the proportion of culture positive cases (67.3%) was relatively higher among patients suffering from blepharitis. Previous report from southern Ethiopia documented higher proportions (84.2%) of culture positive cases among patients suffering from dacrocystitis [33]. Another report from India also documented a significant association of culture-positive bacterial infection among patients with dacrocystitis [34]. On the other hand, conjunctivitis was reported as an ocular infection that showed high association with culture positive bacterial infection of the eye in Nigeria [35].

In this study, the dominant bacterial isolates from external ocular infection were Gram positive cocci. This is supported by several previous reports from Ethiopia [26–28], and countries like Nigeria [35], India [10] and Iran [15] that also reported Gram positive cocci as the primary cause of bacteria caused external ocular infection. In the present study, S*. aureus* was the predominant isolate concordant with previous study reports in the same area [35], Hawassa [22], Jimma [12] and India [6]. Nevertheless, Muluye et al. [27] reported CoNS as the predominant bacterial pathogen among ophthalmic patients in Gondar. The prevalence of CoNS reported by Muluye et al. (27.4%) was lower than that of the current study which is 33.5%. The increase in the prevalence of Gram positive cocci might be due to contamination of the eye from skin normal flora as a result of touching the eyes with contaminated hands.

The data of the present study showed that the predominant bacteria from conjunctivitis cases were *S.aureus* followed by CoNS accounting for 51*.*1% and 31.1%, respectively. In addition, *S. aureus* was the most frequently isolated bacteria (50.6%) followed by CoNS (32.9%) among blepharitis cases. The pathogenic bacteria isolated from a dacryocystitis case were *S.aureus* and CoN*S* with equal frequency. This result is supported by several reports from different parts of Ethiopia and abroad [16, 36]. On the other hand, *S. pneumoniae* was reported as the dominant pathogen among dacryocystis cases in Hawassa [15].

The current study showed higher proportions of the *S. aureus* isolates were resistant to penicillin, ampicillin, amoxicillin, and ceftazidime. This result agrees with previous report from Hawassa and Gondar [15, 26]. On the other hand, the prevalence of MRSA infection among ophthalmic patients in Gondar was 24%. The prevalence of MRSA infection was determined based on the resistance pattern of cefoxitin. The gold standard for identifying MRSA is to detect the *mecA* gene [37], or its product, Penicillin-Binding Protein 2a (PBP2a), by latex agglutination [38, 39]. However, these tests are not within the scope of many clinical laboratories and are relatively expensive. Cefoxitin and moxalactam have been reported as surrogate markers for the detection of methicillin resistance [40, 41]. Previously, one study conducted in UK [42] reported a 3% prevalence of MRSA infection among ophthalmic patients and concluded that MRSA is yet an infrequent cause of external ocular infections. On the other hand, in Japan, Fukuda et al. [43] demonstrated that out of 115 *S. aureus* isolates from patients with bacterial conjunctivitis, 74 (64%) were MRSA. This illustrates the marked variation in the prevalence of MRSA ocular infections geographically and at different time points. Reports of MRSA ophthalmic infections are increasing in the literature. Of special concern to ophthalmic surgeons are the increasing reports of postoperative MRSA infection. Kato and Hayasaka [44] found that 13 of 978 eyes (1.3%) swabbed preoperatively grew MRSA. Genetic analysis of MRSA strains from around the world revealed that transfer of SEC*mec* gene to a MSSA strain occurred only a few times, so the emergence of MRSA resulted from dissemination rather than the development of new MRSA clones [44]. Thus, virtually all patients with MRSA infection or colonization acquire their strain from an external source [45, 46]. The success reported in controlling MRSA by rigorous infection control practices supports the premise that transmission is the major factor contributing to the increasing prevalence of MRSA [47], whereas antibiotic therapy is an important risk factor for MRSA by providing a selective advantage for its survival and spread [46].

The drug susceptibility patterns of Gram negative bacterial isolates showed a 34.8% resistance to tetracycline, 30.4% to chloramphinicol, 21.7% to gentamicin and 56.5% to amoxicillin ‘and’ ampicillin each. The overall MDR pattern of the Gram negative bacterial isolates was 92.1%. The drug susceptibility pattern of *Klebsiella* species showed a 100% resistance to ampicillin and amoxicillin**.** The prevalence of multi-drug resistance to two or more commonly prescribed antimicrobials was 69.9% in Hawassa and the proportion of MDR isolates for *Serratia marcesens*, *Proteus mirablis* and *Klebsiella* species was 50%, 40% and 33%, respectively [15]. A previous report by Muluye et al. also showed [27] higher prevalence of MDR Gram negative bacteria prevalence among ophthalmic patients in Gondar. An irrational use of antimicrobials without prescription, improper dosage regimen, misuse of antimicrobials for viral and other non-bacterial infections, extended duration of therapy, and migration could result in increased antimicrobial resistance.

The limitation of the current study was that *Chlamydia trachomatis*, *Corynebacterium* species and anaerobic bacteria caused ocular infections were not investigated due to resource problems.

## Conclusion

Conjunctivitis was the dominant external eye infection followed by blepharitis among patients at University of Gondar hospital. The prevalence of Gram positive cocci was 88% and the dominant bacterial species was *S. aureus*. The majority of the *S. aureus* isolates were resistant to ampicillin, amoxicillin, and ceftazidime. The prevalence of MRSA infection was 24%, and the overall MDR bacterial pathogen proportion was very high (92.1%). The high prevalence of MRSA and MDR bacterial pathogens dictates that the need for effective prevention is as important as for therapies. Regular face washing could reduce bacteria caused external eye infection for which health education is indispensable.

## Abbreviations

ATCC: American Type culture collection
BAP: Blood agar plate
BHI: Brain heart infusion
CAP: Chocolate agar plate
CI: Confidence interval
CLSI: Clinical and Laboratory Standard Institute
CMHS: College of Medicine and Health Sciences
CoNS: Coagulase negative *Staphylococci*
I: Intermediate
IQR: Inter quartile range
LDC: Lysine decarboxylase
MAC: MaCconkey agar
MDR: Multi-drug resistance
MHA: Muller Hinton agar
MRSA: Methicilline resistant *Staphylococcus aureus*
MSA: Manitol salt agar
MSSA: Methycilline sensitive *Staphylococcus aureus*
OR: Odds ratio
PBP2a: Penicillin-Binding Protein 2a
QC: Quality control
R: Resistant
S: Susceptible
SOPs: Standard operating procedures
SPSS: Statistical Package for Social Sciences
TSI: Triple sugar iron agar
UK: United Kingdom
UOG: University of Gondar
WHO: World Health Organization
